# “BreaThink”: breathing affects production and perception of quantities

**DOI:** 10.1007/s00221-021-06147-z

**Published:** 2021-06-12

**Authors:** Francesco Belli, Arianna Felisatti, Martin H. Fischer

**Affiliations:** Cognitive Sciences Division, Psychology Department, University of Potsdam, Karl-Liebknecht-Str. 24-25, 14476 Potsdam, Germany

**Keywords:** Breathing, Embodied cognition, Interoception, Numerical cognition, Situated cognition

## Abstract

Cognition is shaped by signals from outside and within the body. Following recent evidence of interoceptive signals modulating higher-level cognition, we examined whether breathing changes the production and perception of quantities. In Experiment 1, 22 adults verbally produced on average larger random numbers after inhaling than after exhaling. In Experiment 2, 24 further adults estimated the numerosity of dot patterns that were briefly shown after either inhaling or exhaling. Again, we obtained on average larger responses following inhalation than exhalation. These converging results extend models of situated cognition according to which higher-level cognition is sensitive to transient interoceptive states.

## Introduction

Cognition refers to the mental activities of higher living systems and includes component processes such as attention allocation, stimulus encoding, memory consolidation, knowledge retrieval, motor production, and others (e.g., Ashcraft and Radvansky 2017; Sternberg and Sternberg 2016). These cognitive processes are studied from different theoretical perspectives, among which embodied cognition is currently influential. According to embodied cognition principles, all aspects of human cognition are shaped by their implementation in the human body which provides sensory experiences, enables motor processes and sends interoceptive signals about bodily states to the brain (reviews in Fischer and Coello 2016; Raab 2020; Shapiro 2019). Several behavioral signatures indicate an intricate interconnection between body and mind. For example, we make more lenient decisions after lunch than before, reflecting an influence of blood sugar levels on cognition (Danziger et al. 2011; Gailliot and Baumeister 2007) and we tend to perceive cartoons as funnier while activating smiling- compared to frowning-related muscles, indicating that muscle tension is incorporated into emotional judgments (Noah et al. 2018). According to embodied cognition, even the acquisition and activation of abstract concepts, such as numbers, is crucially influenced by the body (Anobile et al. 2021; Fischer 2012, 2018; Hartmann et al. 2012).

Recently it became evident that also repetitive autonomous bodily activities, such as heart beats and breathing cycles, modulate our cognitive processes (e.g., Critchley and Garfinkel 2018; Park et al. 2020). For example, slower breathing improves performance in a Stroop task (Prinsloo et al. 2013), presumably mediated through cardiac vagal activity (cf. Hoffman and Lumpkin 2018). Memory for briefly presented words is reduced when they are learned during systolic contraction compared to diastolic relaxation of the heart muscle, presumably reflecting interoceptive interference with stimulus encoding due to acutely increased blood pressure (Garfinkel et al. 2013; see also Thayer et al. 2009). Similarly, we initiate voluntary actions more frequently during the exhalation or expiration phase of breathing compared to the inhalation or inspiration phase (Park et al. 2020). Given this recent surge of evidence for an impact of autonomous bodily rhythms on stimulus encoding and action production, as well as an advanced understanding of respiratory control mechanisms (Del Negro et al. 2018; Yackle et al. 2017), we aimed to investigate the impact of our breathing rhythm on higher cognitive processes.

Inhaling oxygen from the atmosphere and exhaling carbon dioxide from the lungs is essential for our bodily existence. Normal respiration in human adults involves 10–12 breaths per minute at rest and this changes their lung volume by up to 5 L (e.g., Del Negro et al. 2018; Hough 2001). The rhythmic process of inhaling and exhaling is controlled through neuronal networks in subcortical brain structures (pons and medulla) that receive input from the spindles in our breathing muscles, stretch receptors in the lungs, arterial pressure receptors and chemoreceptors that monitor the composition of oxygen and carbon dioxide in the blood (Del Negro et al. 2018; Scheid 2005). Breathing involves also motoric and limbic structures (e.g., the hypothalamus) and generates peripheral pressure and stretch signals that convey multimodal information to the central breathing control system.

There is a long tradition of modulating mental states through breathing (e.g., Fried and Grimaldi 1993). For example, inhaling reduces and exhaling increases interoceptive signaling from the body to the brain via the vagus nerve (Gerritsen and Band 2018). While the anterior insula is a convergence point for interoceptive signals in the brain (Allen 2020; Craig 2009), quantitative or intensity information from all senses converges in the parietal lobes and this has sparked A Theory of Magnitude (ATOM; Bueti and Walsh 2009; Walsh 2003, 2015) according to which the parietal lobes house a generalized or cross-modal magnitude system. Although ATOM currently makes no reference to interoceptive intensities or magnitudes, it explains a wide range of cross-domain interactions between stimulus quantities, including time, space, weight and numerosity. Here, we explored whether the production of quantities (Experiment 1) and the encoding of quantities (Experiment 2) depend on interoceptive breathing signals by manipulating participants’ respiratory state.

## Experiment 1: quantity production

To assess whether breathing-related interoceptive signals influence the production of quantities, we adopted the random number generation (RNG) task. This task measures general cognitive capacity, such as executive functions of working memory (Baddeley 1986). RNG performance exhibits several systematic signatures, such as a tendency to avoid repetitions and to produce counting-like responses in demanding situations (e.g., Brugger 1997). Moreover, RNG has already proven sensitive to repetitive bodily changes, leading to larger numbers on average being stated following rightward or upward compared to leftward or downward head movements (Loetscher et al. 2008; Winter and Matlock 2013). Interestingly Loetscher and colleagues (2010) demonstrated how even the direction of the eyes is a predictor of the magnitude of the subsequently generated number. Similarly, Audiffren and colleagues (2009) described that exercise led to longer ascending and descending series of RNG compared to a resting condition. To our knowledge there has been no published work on interoceptive effects on RNG. Given that exercise requires more and deeper breathing (Casaburi et al. 1989; Courteix et al. 1997), we expected to see systematic effects of inhalation vs. exhalation on RNG.

### Method

#### Participants

Twenty-six voluntary participants (13 females, 13 males) naive to the task and to the hypothesis (mean age 27 years, SD 5, range 18–43) were tested, comparable with sample sizes used in similar studies (Hartmann et al. 2012; Shaki and Fischer 2013; Sosson et al. 2018). The participants had different native languages (11 Italians, seven Turkish, seven Germans, one Japanese) and were recruited at the University of Potsdam and among friends and relatives of the researchers. All participants were treated in accordance with ethical guidelines as laid down in the Declaration of Helsinki.

#### Procedure

As a result of the outbreak of the corona virus pandemic during the time of testing, participants were tested in two settings: 13 through individual on-line video calls, the other 13 in person at the lab. Participants were instructed to sit comfortably on a chair in a quiet room, put their hands on their legs, close their eyes and breathe deeply and regularly through their nose or mouth.

Each participant was told to produce 40 random numbers between 1 and 10, alternatingly after each successive deep inhalation and deep exhalation (i.e., two random numbers per breathing cycle), in their mother tongue. They were asked to produce each number at the very end of each breathing process and to be as random as possible. To explain the concept of “randomness”, the experimenter instructed each participant to “imagine picking up a number out of a hat, returning it, put it back in the hat, shaking the hat's contents, then picking another number out of the hat, and so forth” (cf. Audiffren et al. 2009; Horne et al. 1982; Jahanshahi and Rothwell 2000). Before data collection, participants were briefly trained on how to correctly perform this task (see Appendix 1).

The initial breathing state when generating the first random number (start with inhaling vs. start with exhaling) was counterbalanced between participants. The experimenter had full frontal view of each participant and noted down every number produced. No number-related feedback was given to the participants during training or data collection. At the end of the testing phase, participants were asked to give feedback and report whether they encountered any difficulty during the experiment. No difficulty was reported. Instruction, data collection (40 random numbers per person in total) and debriefing lasted approximately 10–15 min.

### Analyses

Analyses were performed using Microsoft Excel, IBM SPSS Statistics 26 and JASP v0.11.1.0. Four participants were excluded from the analysis: Two participants for massive delays and instable internet connection compromising the reliability of the task; two participants due to interruptions during data collection (opening the eyes and asking questions; other person entering the room). All remaining responses were in the prescribed numerical range and therefore were included in the analyses. Raw data are available at http://doi.org/10.17605/OSF.IO/6WQAB.

**Fig. 1:**
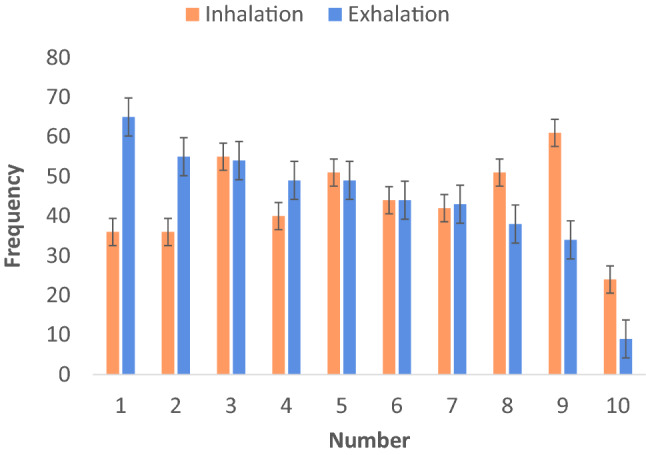
Frequencies of random numbers produced after inhalation (orange) vs. exhalation (blue). Error bars indicate ± 1 standard error of the mean

**Fig. 2:**
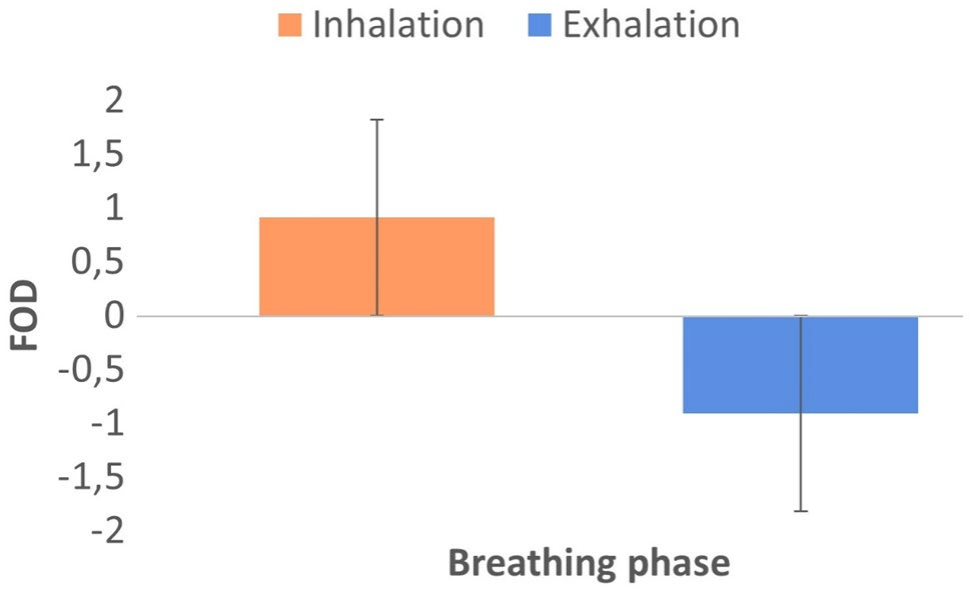
Average of first order differences (FOD) after inhalation (orange) vs. exhalation (blue). Error bars indicate ± 1 standard error of the mean

We performed the following analyses on data from the remaining 22 participants (11 females, 11 males). Post-hoc determination of power for *t*-testing revealed a 73% chance of correctly rejecting the null hypothesis of no significant effect with a total of 22 participants, given an expected medium effect size and *p* < 0.05. First, we counted the frequencies of numbers chosen for every participant (see Fig. 1) and then calculated the averages of generated numbers for every breathing phase (inhalation, exhalation) for each participant. Consistent with ATOM (Walsh 2003, 2015), we predicted larger values for all these descriptive statistics following inhalation compared to exhalation. We also assessed the quality of participants’ randomness by computing First Order Differences (FODs; Sosson et al. 2018; Towse and Neil 1998) and Redundancy scores (R scores; Towse and Neil 1998). FODs refer to the difference between each number and its preceding number. An FOD value of 0 implies a repetition of the preceding number and is known to be rare in human RNG, while a value of 1 reflects counting and is known to reflect increased cognitive load. Consistent with ATOM (Walsh 2003, 2015), we expected to observe a stronger ascending trend (mean FOD > 0) after inhalations and a stronger descending trend (mean FOD < 0) after exhalations (see Fig. 2). R scores reflect the ability of the participant to be random. In particular, the lower the R scores, the higher the randomness is (R score equal to 100% indicates total redundancy and, as a consequence, null randomness).

Mixed-factors analyses of variance (ANOVA) evaluated the effects of breathing phase (inhalation vs. exhalation) as within-subject factor and setting (video call vs. laboratory) and starting condition (start with inhaling vs. start with exhaling) as between-subject factors on both dependent measures. We used Greenhouse–Geisser corrections whenever sphericity assumptions were violated.

### Results

#### Average random numbers generated

There was a reliable main effect of breathing phase [*F*(1, 18) = 16.59; *p* < 0.001; ${\eta }_{p}^{2}$ = 0.48], with a mean of 5.55 after inhalation and 4.64 after exhalation. There were no reliable main effects of either starting condition [*F*(1, 18) = 0.08; *p* = 0.788; ${\eta }_{p}^{2}$ < 0.01] or setting [*F*(1, 18) = 1.05; *p* = 0.318; ${\eta }_{p}^{2}$ = 0.05] and no significant interactions between these factors (all *p* values > 0.05). Paired samples t-tests on extreme number segments (occurrences of numbers 1–2 and 9–10) and medium number segments (occurrences of numbers 3–4–5 and 6–7–8) in both conditions (after inhalation and after exhalation) showed a reliable effect of breathing on extreme magnitudes for the segment 1–2 [*t*(21) =  − 3.5203, *p* = 0.002] and the segment 9–10 [*t*(21) = 4.713, *p* < 0.001]. No main effect from breathing emerged by *t*-tests on the medium segments (all *p* > 0.05).

#### First order differences

There was a reliable main effect of breathing phase [*F*(1, 18) = 17.84; *p* < 0.001; ${\eta }_{p}^{2}$ = 0.50] with a mean of + 0.91 after inhalation and a mean of − 0.91 after exhalation. There were again no main effects of starting condition [*F*(1, 18) = 0.88; *p* = 0.362; ${\eta }_{p}^{2}$ = 0.04] or setting [*F*(1, 18) = 0.88; *p* = 0.362; ${\eta }_{p}^{2}$ = 0.04] and no interactions (all *p* > 0.05).

#### Redundancy score

Paired samples t-test analysis on R scores revealed no main effect of breathing phase [*t*(21) = 0.026, *p* = 0.980], with participants scoring on average *R* = 12.31% after exhalations and 12.28% after inhalations. This result reflects good compliance on the random number generation task in both respiratory phases, confirming the effect of breathing on the numerical magnitude productions and not on the ability to be random.

### Discussion

Our first experiment established the novel finding that the production of numbers is affected by respiratory state. Participants generated more large numbers such as 9 or 10 following inhalation and more small numbers such as 1 or 2 following exhalation (see Fig. 1). This previously uncontrolled aspect of performance in the widely used RNG task was predicted from an embodied cognition perspective according to which all cognitive performance is affected by bodily signals. Some previous RNG studies relied on simple correlations between body states and numerical cognition (Loetscher et al. 2008, 2010; Winter and Matlock 2013), while our first experiment provided evidence for a causal link between bodily signals and numerical magnitude production.

Before discussing this finding and its theoretical implications, some methodological concerns should be addressed. First, all verbal productions require some exhalation because the air stream from the lungs must vibrate our vocal chords (Rosenbaum 2009, chap. 10). Thus, all responses in our RNG task were given while exhaling (although with vastly different quantities of air in the lungs) and this may underestimate the true effect size for the breathing-related impact on quantity processing, currently estimated at ${\eta }_{p}^{2}$ = 0.48–0.50. The described effect was observed in 17 participants out of 22. Therefore, we next presented visual quantities very briefly either at the end of inhalation or at the end of exhalation to obtain a more valid estimate for the size of the breathing-related effect on quantity processing. This improved method would also extend our discovery to the perceptual domain, consistent with a prediction from embodied cognition, according to which also encoding-related processes are affected by bodily signals (e.g., Abrams et al. 2008; Fischer and Hoellen 2004; Symes et al. 2008). Finally, in light of the current reproducibility crisis (e.g., Camerer et al. 2018) a conceptual replication of our novel finding was desirable.

## Experiment 2: quantity perception

In order to assess whether breathing-related interoceptive signals influence the perception of quantities, we adopted the visual numerosity estimation task. This task is widely used to study quantitative abilities in both humans and animals (e.g., Brannon and Merritt 2011; Dehaene et al. 1998; Krueger 1972) and predicts arithmetic abilities in children (Halberda et al. 2008). The numerosity of dot patterns exceeding six dots (the so-called subitizing range) is generally underestimated (Izard and Dehaene 2008). Consistent with embodied cognition, sensory features that are normally correlated with numerosity, such as the area, density, and convex hull of a dot cloud, also influence perceptual judgments (DeWind et al. 2015). To our knowledge there has been no published work on interoceptive effects on numerosity perception.

### Methods

#### Participants

Twenty-seven new voluntary participants (20 females, seven males) naive to the task and to the hypothesis (mean age 27.7 years, SD 8.8, range 19–55) were tested. The participants had different native languages (12 Germans, six Italians, three English, two Turkish, one Greek, one Indian, one Persian, one Spanish) and were recruited at the University of Potsdam through an on-line recruiting platform. They were treated in accordance with ethical guidelines as laid down in the Declaration of Helsinki.

#### Stimuli

Four different patterns of 12, 24, 36 and 48 yellow dots on black background were created with Matlab. The size of every single dots was eight pixels and the minimal separation between dots was 3 mm; area, density, and convex hull co-varied with numerosity because we were interested in establishing a new finding under the most natural conditions. Stimuli were shown in four different orientations (0, 90, 180 and 270°), resulting in 16 distinct images. Materials are available at: http://doi.org/10.17605/OSF.IO/6WQAB

#### Procedure

Reflecting the corona virus pandemic, participants were again tested in one of two settings: either on-line or in person at the lab. Participants were instructed to sit comfortably on a chair in front of a PC monitor, put their hands on their legs and to breathe deeply and regularly. The breathing state when generating the random numbers (after inhaling vs. after exhaling) had to be blocked to prevent confusion on the part of participants and block order was counterbalanced between participants.

Beginning with their assigned starting condition, participants had to close their eyes when they saw a small white fixation cross at the center of the screen. Then they had to begin their breathing cycle and open their eyes again when reaching the instructed stimulus encoding state, which was either the peak of inhalation or the peak of exhalation. This eye opening was the signal for the experimenter to present one of the 16 stimuli, randomly chosen by PsychoPy software and displayed for 500 ms. Immediately following this, the screen turned blank and the participants verbally estimated the quantity of dots they had perceived and waited for the next fixation cross to continue with the experiment. Each participant completed two blocks of 16 trials (stimulus encoding at peak inhalation, stimulus encoding at peak exhalation).

Participants were instructed to respond as fast and accurate as possible without using strategies such as counting. Before data collection, participants were briefly trained on how to correctly perform this task (see Appendix 1). The experimenter either sat in front of the participant or (during on-line testing) had full frontal view of the participant and noted every estimate on a pre-prepared response sheet. No other person was present during testing and no number-related feedback was given to the participants during training or data collection. During the debriefing phase at the end of the experiment, participants were asked to give feedback and report whether they encountered any difficulty during the experiment. No difficulty was reported. Instruction, data collection (32 trials per person in total) and debriefing lasted approximately 15 min.

### Analyses

All analyses were performed using Microsoft Excel, IBM SPSS Statistics 26 and JASP v0.11.1.0. Three participants were excluded from the analysis: one person produced several extreme outlier numbers (e.g., 200, 250), suggesting that she did not correctly understand and perform the task; the other two participants were excluded because of instable internet connections during on-line testing, resulting in large transmission delays that compromised the reliability of responses. We performed the analyses on data from 24 participants (17 females, seven males). Raw data are available at http://doi.org/10.17605/OSF.IO/6WQAB. Post-hoc determination of power for ANOVA revealed a 99% chance of correctly rejecting the null hypothesis of no significant effect with a total of 24 participants, given an expected medium effect size and *p* < 0.05.

**Fig. 3:**
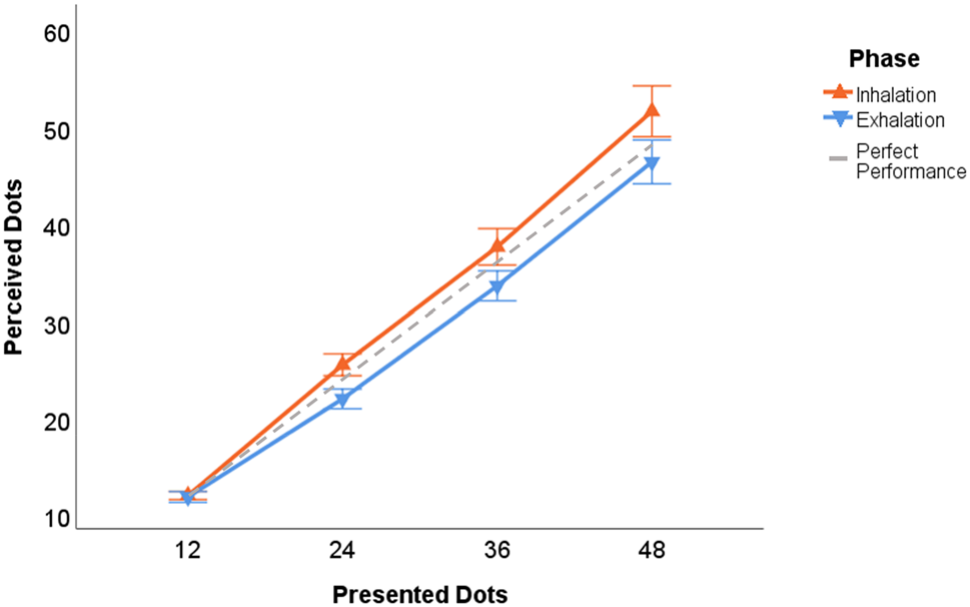
Estimates of dot numerosities given after inhalation (orange line) or exhalation (blue line). Dashed line indicates perfect performance. Error bars represent ± 1 Standard Error of the mean

We calculated mean estimates for each numerosity in every breathing phase (inhalation, exhalation) for each participant and plotted estimated against actual numerosities (see Fig. 3). Then we fitted linear regressions to obtain slope and intercept values and assess task compliance. Average slopes in this task can range from 0.7 to 1.3 for healthy adults (Krueger 1972), with one indicating perfect performance. Intercepts were computed to assess possible breathing effects unrelated to the numerosity manipulation (cf. Lachman et al. 1979, p. 155). Based on Walsh (2003, 2015) and Bueti and Walsh (2009) we predicted larger slope and/or intercept values following inhalation compared to exhalation.

An initial mixed-factors ANOVA with breathing phase (inhalation vs. exhalation) as within-subject factor and with setting (video call vs. laboratory) and starting condition (start with inhaling vs. start with exhaling) as between-subject factors showed no significant effects on mean estimates relating to the between-subject factors. Thus, a repeated-measures ANOVA evaluated effects of breathing phase (inhalation vs. exhalation) and numerosity (number of dots) on mean estimates. We used Greenhouse–Geisser corrections whenever sphericity assumptions were violated.

### Results

#### Average numerosity estimates

The analysis of mean estimates revealed a reliable main effect of breathing phase [*F*(1, 23) = 22.03; *p* < 0.001,${\eta }_{p}^{2}$ = 0.49], with larger overall estimates after inhalation (31.75) compared to exhalation (28.52). Aside from the (trivial) main effect of numerosity [*F*(1, 23) = 242.67, *p* < 0.001, ${\eta }_{p}^{2}$ = 0.91] there was also a reliable interaction between breathing phase and numerosity [*F*(1, 23) = 6.40; *p* = 0.003,${\eta }_{p}^{2}$ = 0.22]. The estimates for 12, 24, 36 and 48 dots differed by 0.16, 3.51, 4.02, and 5.23 dots for inhaling compared to exhaling, amounting to 1.3, 14.6, 11.2, and 10.9% overestimation, respectively. The three largest percentage scores differed reliably from zero, with all *p*-values < 0.001, but not among each other, with all *p*-values > 0.07.

#### Slopes and intercept

There was a reliable main effect of breathing phase on the regression slopes [*F*(1, 23) = 6.934; *p* = 0.015, ${\eta }_{p}^{2}$ = 0.23], with larger slopes after inhalation (1.08) compared to exhalation (0.97). Nevertheless, in both breathing states performance was overall accurate, as indicated by non-reliable *t*-tests against unity for inhalation [*t*(23) = 1.135, *p* = 0.27] and exhalation [*t*(23) =  − 0.521, *p* = 0.61], respectively. There was no reliable main effect of either starting condition or setting and no significant interactions between the factors (all *p* values > 0.05).

The average intercept was  − 0.49. There was no reliable main effect of breathing phase on the intercepts [*F*(1, 23) = 0.004; *p* = 0.952, ${\eta }_{p}^{2}$ = 0.002] and no reliable main effects of either starting condition or setting and no significant interactions between the factors in both analyses (all *p* values > 0.05).

### Discussion

Our second experiment replicated the main finding from Experiment 1 in a novel task: adult participants perceived dot patterns to be more numerous when shown after inhalation than when shown after exhalation. Although all numerosities were above the subitizing range the breathing bias only emerged for numerosities larger than 12, which suggests that breathing-related signals are only incorporated into numerosity estimates when uncertainty exceeds a minimum. The breathing-related errors for larger numerosities comply with Weber’s Law as they are proportional to the stimulus (Dehaene et al. 1998; Gallistel and Gelman 1992). The overall effect size was similar to that of Experiment 1, with 20 participants out of 24 showing the effect. This indicates that exhaling during verbal productions in our first experiment did not diminish the effect. Having addressed this concern and replicated the main finding, we now turn to a broader discussion.

Finally, the effect on the quantity perception observed in Experiment 2 could be a result of covariance of perceptual aspects and properties (such as overall area, density and convex hull of dots) which were not controlled in this study.

## General discussion

The present study investigated systematic effects of breathing signals on cognition. It was inspired by an influential theoretical proposal in the framework of embodied cognition, according to which quantity-related processing converges on a common neural representation (Bueti and Walsh 2009; Walsh 2003, 2015). We collected two types of quantitative responses, namely randomly produced numbers and estimates of visually perceived numerosities, after participants had either just inhaled or just exhaled. Consistent with the proposal, inhalation and exhalation caused larger and smaller responses, respectively, in both numerical production and perception. Indeed, the observed effect size was substantial and consistent across tasks. This novel finding has both theoretical and practical implications which we will discuss after speculating about its origin.

How does this breathing-related cognitive bias come about? Respiration is largely regulated by the autonomous central nervous system through respiration control centers within the brain stem, specifically in the pons and medulla. However, we do have some voluntary control over breathing through motor-cortical activity that possibly contributes to cognitive tasks, e.g., via action simulation (Paccalin and Jeannerod 2000). Thus, we consider in the following both bottom-up and top-down mediation.

One obvious candidate for a bottom-up mediation of breathing effects on quantitative cognition is the re-oxygenation of a person’s blood following inhalation. This increased blood oxygenation will eventually be registered in the brain where oxygen is transported to those areas specifically contributing to the cognitive task at hand. However, given that the time-course of the blood–oxygen-level-dependent (BOLD) response in functional magnetic resonance imaging is in the order of 5 s (Chang and Glover 2009; Logothetis and Wandell 2004), the effect of inhaling on cortical blood oxygenation would be too sluggish to account for the observed biases in the immediate responses delivered by our participants in both tasks. Nevertheless, the inter-trial interval between successive assessments should be systematically varied in future studies to prevent a coupling of the breathing-related BOLD component with stimulus presentations.

Alternatively, consider stretch signals in the lung tissue and spindles of the breathing muscles and their afferent signaling via the vagus nerve (Del Negro et al. 2018; Gerritsen and Band 2018) as another candidate mechanism for the bottom-up mediation of the present results. Specifically, increased biomechanical pressure might bias higher-level cognition to signal more stimulation, which then induces the observed perceptual and production biases as the result of convergence of this and other signals in some central processing stage as proposed by ATOM (Walsh 2003, 2015; see also Craig 2009).

However, inhaling is known to actually *reduce* afferent vagus nerve activity (Chang et al. 2015), which conflicts with this hypothesized mechanism of action to the extent that breathing signals were relayed along this pathway. Moreover, given that inspiration tends to increase heart rate (Perry et al. 2019), this proposal also conflicts with work by Garfinkel et al. (2013) and Critchley and Garfinkel (2018). Those authors showed that increased blood pressure from systolic heart contractions *reduces* perceptual sensitivity and inhibits memory recall when encoding items at systole but not at diastole.

Consider now a possible top–down mediation of the breathing effect on quantitative processing; specifically, the notion that bodily signals such as postural and muscular proprioception affect concept availability (Andres et al. 2004; Dijkstra et al. 2007) and valence judgments (Danziger et al. 2011; Noah et al. 2018). We note that inhaling and exhaling slightly raises and lowers one’s posture, respectively. This systematic postural change might activate conceptual metaphors of “more is up” and “down is less” that are known to influence signatures of cognitive embodiment (Carney et al. 2010; Fischer 2012).

Relatedly, it is possible that the breathing effect we report here is also partially mediated through linguistic associations, such as “inhaling is more” or “exhaling is less”, consistent with the corresponding changes in lung volume. Given that our tasks induced response uncertainty on the part of participants, decision heuristics may also have contributed to the observed breathing bias (Kahneman 2013). Our tasks, as well as our hypotheses, may have been transparent to participants and we failed to examine their predictions about the purpose of our study after data collection. Future studies should assess and control possible demand characteristics, as well as the contribution of linguistic factors to our results.

We turn now to potential theoretical implications of our finding. First, the breathing effect documented in our study supports a common representation of all kinds of sensory magnitudes in our brain. This contribution of breathing to magnitude processing calls for an extension of the scope of ATOM (Walsh 2015) to encompass also interoceptive signals. Indeed, inhalation relates to larger magnitudes and exhalation to smaller ones, and this covariation extends across perception and production. A similar proposal to extend the scope of ATOM was recently made by Miklashevsky et al. (2021) who reported spontaneous grip force adjustments after number processing. ATOM is concerned with spatial and action-related magnitudes; while our hands are prime candidates for voluntary action, breathing is a less controlled or intentional act but still related to action initiation (Park et al. 2020). Future neurocognitive studies should determine the neurophysiological substrate of this interoceptive effect by targeting connections between the vagus nerve and the two afferent integration centers anterior insula and parietal cortex (Gasquoine 2014; Vonck et al. 2014).

Breathing-related body signals fluctuate several times per minute. Within the broader framework of numerical cognition, such transient influences on cognition have been theorized by Fischer (2012) who distinguished situated from embodied and grounded number knowledge. In this view, *grounded* cognition refers to evolutionarily inherited physical constraints, such as the impact of gravity or object impermeability on spatial-numerical associations, while *embodied* cognition acknowledges the impact of individual and culturally shaped sensori-motor learning histories on cognition. Importantly, *situated* factors such as recently performed attention shifts (Fischer et al. 2009) and recently encountered spatial-numerical associations (Fischer et al. 2010) are particularly powerful: they can dilute and even reverse such associations. The present findings of strong breathing-related changes (with effect sizes of around 0.50) in numerosity perception and production fit well with this theoretical position.

This work will also have practical implications once it has been substantiated by convergent results across cognitive domains. Previous cognitive assessments left effects of participants’ breathing on their performance largely uncontrolled because the classical notion of human cognition as computation does not encompass interoceptively mediated effects. Yet, our observations document a direct link between breathing and higher-level cognition, thus constituting clear evidence for the embodied nature of cognition. Our study brings important practical implications in support of meditative and therapeutical practices such as mindfulness and pranayama (Bing-Canar et al. 2016; Melnychuk et al. 2018), where the link between respiration and cognition plays a crucial role.

Consider as an applied example the domain of numerical cognition. We found signatures of breathing-related processes on two widely used numerical tasks: random number generation is a standard method to examine or manipulate a person’s mental load (e.g., Baddeley et al. 2015; Brugger 1997) while numerosity estimation is used to predict mathematical achievement (e.g., Halberda et al. 2008; Schneider, 2017). Our results show that both tasks are influenced by interoceptive signals (i.e. breathing). In turn, controlling this source of variability will result in more precise cognitive assessments.

### Limits of the study and future directions

To keep the methodology of the experiments as simple as possible, we could not avoid some limitations that we are now going to describe. The biggest limitation of the study comes from the Covid-19 pandemic outbreak, which forced us to move the study online and face the limits of controlling breathing phases from afar. This resulted in a lack of precise control and the impossibility to record the respiratory phase of the participants. Improvements and suggestions to the design of the present experiments should address most of the current limitations. As claimed before, one of the limits of the study involves the lack of control and recording of the respiratory phases. We, therefore, suggest to improve further investigations by using online monitoring sensors as provided by respiratory belts, with the hope that in the near future this methodology would be allowed again without any risk for participants’ and researchers’ health.

Concerning the production experiment, first the generation rate should be measured since it is an important factor influencing RNG (Brugger 1997). Second, the observations in Experiment 1 revealed the presence of a clear small-number bias (SNB; Loetscher and Brugger 2007) in the exhalation phase, but this bias was not present after inhalations. SNB is an indicator of asymmetric cognitive frontal lobe functions (Bachmann et al. 2010). Considering this, it would be interesting to investigate the connection with hemispheric processing. Future experiments could manipulate which nostril is dominant at the moment of testing in order to investigate contralateral hemispheric activation, as shown by studies on forced unilateral nostril breathing (Block et al. 1989; Jella and Shannahoff-Khalsa 1993).

Further improvements could come from considering non-verbal responding, either per button pressing or per finger posture generation. Using manual instead of verbal responses could help in solving the potential conflict between inhaling and speaking. The present study had minimized motor output to avoid motor biases and thereby established a dataset comparable with the RNG literature where verbal responding is the gold standard.

More generally, future experiments could track the time course of the breathing effect on all aspects of thinking by presenting stimuli or collecting responses at precisely defined moments after inhalation or exhalation, e.g. by utilizing breathing belts. Lab-based studies can also apply mental chronometry to further examine the generality of the effect across tasks and modalities.

## Data Availability

Data and materials are publicly available at http://doi.org/10.17605/OSF.IO/6WQAB.

## Appendix 1

### Statements

- We did not instruct/ask participants to take a pause between the two different breathing phases while performing the RNG task. The RNG production occurred in a continuous flow (i.e., inhalation, number, exhalation, number, inhalation, number… and so on).
- We did not instruct/ask participants to hold their breath.
- We did not instruct/ask participants to produce numbers in sets of two. If they did so during training then this was discouraged.

### Experiment 1, instructions spoken to participants

- Please sit comfortably and put your hands on your legs.
- You will be asked to take deep breathings, deep inhalations and deep exhalations, as deep as you can.
- Then you will be asked to produce a random number from 1 to 10 after each breathing phase, starting accordingly to your assigned starting condition.
- Imagine to have a hat with ten pieces of paper inside, on each piece of paper there is a number from 1 to 10. Picture to reach into the hat for one of those piece of paper, read out loud the number on it and then put the piece of paper back into the hat.
- You will perform this after each inhalation and after each exhalation, so the task will be like this: inhalation, number, exhalation, number… and so on until the researcher will stop you at the end of the task.
- Keep your eyes closed while performing the task and try to avoid interruptions and distractions.
- If you have any question or doubt on how to perform this task, please ask before starting the experiment.

### Experiment 2, on screen instructions

- When you see a fixation cross please close your eyes and take a deep INHALATION/EXHALATION (according to assigned starting condition).
- At the very peak of your inhalation/exhalation (according to assigned starting condition) open your eyes.
- A pattern of random dots will appear for a very short time.
- You will be asked to estimate how many dots you perceived BEFORE starting a new breathing phase.
- So to summarize, the task will go like this: fixation cross, close your eyes, take a deep inhalation/exhalation, peak reached, open your eyes, estimate the dots pattern and so on.
- Please be as accurate and fast as possible in the estimation.
